# Lymphatic Filariasis Elimination Status: *Wuchereria bancrofti* Infections in Human Populations after Five Effective Rounds of Mass Drug Administration in Zambia

**DOI:** 10.3390/tropicalmed8070333

**Published:** 2023-06-22

**Authors:** Belem Blamwell Matapo, Evans Mwila Mpabalwani, Patrick Kaonga, Martin Chitolongo Simuunza, Nathan Bakyaita, Freddie Masaninga, Namasiku Siyumbwa, Seter Siziya, Frank Shamilimo, Chilweza Muzongwe, Enala T. Mwase, Chummy Sikalizyo Sikasunge

**Affiliations:** 1 School of Public Health, University of Zambia, Ridgeway Campus, Lusaka P.O. Box 50516, Zambia; 2 World Health Organization, Corner Andrew Mwenya/Beit Road, Lusaka P.O. Box 32346, Zambia; 3 School of Medicine, University of Zambia, Ridgeway Campus, Lusaka P.O. Box 50516, Zambia; 4 School of Veterinary Medicine, University of Zambia, Great East Road Campus, Lusaka P.O. Box 32379, Zambia; 5 Ministry of Health Headquarters Ndeke House, Lusaka P.O. Box 30205, Zambia; 6 Michael Chilufya Sata School of Medicine, Copperbelt University, Ndola P.O. Box 71191, Zambia

**Keywords:** lymphatic filariasis, pre-TAS, prevalence, antigenaemia, microfilariae, Zambia

## Abstract

Lymphatic filariasis (LF), also commonly known as elephantiasis, is a neglected tropical disease (NTD) caused by filarial parasites. The disease is transmitted via a bite from infected mosquitoes. The bites of these infected mosquitoes deposit filarial parasites, *Wuchereria* or *Brugia*, whose predilection site is the lymphatic system. The damage to the lymph system causes swelling in the legs, arms, and genitalia. A mapping survey conducted between 2003 and 2011 determined LF as being endemic in Zambia in 96 out of 116 districts. Elimination of LF is known to be possible by stopping the spread of the infection through large-scale preventive chemotherapy. Therefore, mass drug administration (MDA) with diethylcarbamazine citrate (DEC) (6 mg/kg) and Albendazole (400 mg) for Zambia has been conducted and implemented in all endemic districts with five effective rounds. In order to determine whether LF prevalence has been sufficiently reduced to levels less than 2% antigenemia and less than 1% microfilaremia, a pre-transmission assessment survey (pre-TAS) was conducted. Therefore, post-MDA pre-TAS was conducted between 2021 and 2022 in 80 districts to determine the LF prevalence. We conducted a cross-sectional seroprevalence study involving 600 participants in each evaluation unit (EU) or each district. The study sites (sentinel and spot-check sites) were from districts that were the implementation units (IUs) of the LF MDA. These included 80 districts from the 9 provinces. A total of 47,235 people from sentinel and spot-check locations were tested. Of these, valid tests were 47,052, of which 27,762 (59%) were females and 19,290 (41%) were males. The survey revealed in the 79/80 endemic districts a prevalence of *Wb* antigens of 0.14% and 0.0% prevalence of microfilariae. All the surveyed districts had an optimum prevalence of less than 2% for antigenaemia, except for Chibombo district. The majority of participants that tested positive for *Wuchereria bancrofti (Wb)* Antigens (Ag) were those that had 2, 3, and 4 rounds of MDA. Surprisingly, individuals that had 1 round of MDA were not found to have circulating antigens of *Wb.* The study showed that all the surveyed districts, except for Chibombo, passed pre-TAS. This further implies that there is a need to conduct transmission assessment surveys (TASs) in these districts.

## 1. Introduction

Lymphatic filariasis (LF), also known as “elephantiasis” is a deforming and disabling disease that is caused by roundworm parasites of the genera *Wuchereria* and *Brugia* that are transmitted by mosquitoes [1,2]. It is an infection of the human lymphatic system by filarial worms [3]. 

In Africa, the common filarial worm which causes this disease is the species *Wuchereria bancrofti* [4,5]. In the Western Province of Zambia, the disease is locally known as “Mbumba” by the Lozi people, whereas the Nsenga of the Eastern province call it “Msakasa”, and the Tumbuka call it “Mchecha” or “Vimba”, which literally means “swelling”. The disease is characterized by swelling (edema) mainly of the lower limbs with thickening of the skin and underlying tissues. Elephantiasis only results when the parasites (worms) lodge in the lymphatic system [6,7]. The swelling develops when the adult worms cause a partial or complete blockage of the flow of lymph, the fluid which drains the tissues and flows in the lymphatic vessels. 

Most surveys in Zambia on *Wuchereria bancrofti* assessed antigenaemia prevalence. A report exists of an incidental finding of three *Wuchereria bancrofti* microfilariae (Mf) cases out of 459 samples tested during the course of searches for other parasites such as malaria or trypanosomes in Northern Rhodesia (present Zambia) in 1946 [8]. *Wuchereria bancrofti* Antigens were identified in 8.6% of tested individuals, and LF microfilariae were identified in 10.9% of circulating filarial antigens (CFA) positive individuals which was 0.9% of all tested individuals in a study conducted in Luangwa district of Zambia [9]. 

Lymphatic filariasis is targeted for elimination as a public health problem by the year 2030 through the treatment of entire populations at risk with repeated annual MDA [10]. Essential for program success is defining and confirming the appropriate endpoint for MDA when the transmission is presumed to have reached a level low enough that it cannot be sustained even in the absence of drug intervention [11]. Guidelines advanced by World Health Organization (WHO) call for a pre-transmission assessment survey (pre-TAS) to determine whether LF prevalence has been sufficiently reduced to levels less than 2% of antigenemia and less than 1% of microfilaremia after, at least, five effective rounds of annual treatment.

The Global Programme to Eliminate Lymphatic Filariasis is the largest public health intervention program attempted to date through MDA [12]. It is worth noting that MDA does not cure filarial infections, but it can reduce or interrupt transmission of new infections by clearing larval parasites from human blood so that they are not available for mosquitoes, which are vectors. 

Evaluation of the MDA is necessary to determine whether the program has achieved its objective of reducing levels of LF microfilariae in endemic populations to an extent where transmission is likely no longer sustainable. Programmes must be able to assess whether MDA has succeeded in lowering the prevalence of infection to a level where recrudescence is unlikely to occur [13]. Thus, TASs are designed to help program managers determine whether areas have reached this critical threshold of infection [14]. While the TAS provides helpful evidence to national programs regarding the decision to stop MDA, program managers must thoughtfully consider the decision about whether to stop or continue MDA.

## 2. Materials and Methods

### 2.1. Study Design and Population

Using data from a cross-sectional seroprevalence community-based study conducted in eighty (80) evaluation units (EUs), we estimated the seroprevalence in Zambia. The administrative/geographic district which was an implementation unit (IU) for MDA was designed to be an EU with two study sites each: a sentinel site and a spot-check site, which was designated as a control site. The study enrolled persons who were aged five years and above at the time of the fifth MDA round. Each of the sentinel or spot-check sites was targeted to enroll 300 participants, totaling 600 participants per district. The MDA was administered annually for five (5) years in every endemic district. All the districts achieved an effective coverage of 65% and above.

The study setting regarding target population and geographic scope is that Zambia is administratively divided into 10 provinces and 116 districts. The 80 out of 96 endemic districts were due for pre-TAS implementation while 16 districts in Western Province would be conducted upon completing five MDAs rounds. The 80 endemic districts’ MDA target population represented 66% of the estimated country population in the year 2022. The pending 16 endemic districts, in Western Province of Zambia, have planned to conduct pre-TAS during the year 2023.

### 2.2. Sampling Strategy

The study sites (sentinel and spot-check sites) were from districts that were the IUs where MDA, a preventative chemotherapy against LF, was conducted. These include 80 districts from the 9 provinces that were surveyed, namely: Central, Muchinga, Eastern, Copperbelt, Northwestern, Southern, Lusaka, Northern and Luapula provinces.

### 2.3. Timeline of the Survey

The LF mapping data was collected prior to the first round of the MDA 2003 to 2011 [15,16]. Antigenemia (Ag) prevalence was determined using an immunochromatographic test (ICT), which is a testing kit for *W. bancrofti* antigens [17]. However, the countrywide mapping prior to MDA did not assess LF microfilariae prevalence. The five rounds of MDA were implemented between 2014 and March 2021 targeting the population aged 2 years old and above. 

The standard timeline used for the pre-TAS(s) is 6 months after the 5th round of LF MDA with effective coverage [18]. Owing to the large exercise of reaching the 80 districts in 9 provinces, the pre-TAS was conducted in two phases in December 2021 and July 2022

### 2.4. Determination of the Prevalence of LF Antigen

The Alere^TM^ Filarial Testing Strips (FTS) were used to test for LF antigen. The middle or index finger of consenting individuals was cleaned using a cotton ball soaked in 70% alcohol. After drying, the tip of the finger was pricked using a sterile lancet, and blood was immediately collected using capillary tubes and loaded on a sample pad for the FTS test. The results were then read by a trained timekeeper after exactly 10 min. The test requires only 75 µL of blood to be collected from each participant and added to the card which gives a result in 10 minutes while still in the field. Thus, the 75 µL of blood drawn from each participant is placed on an FTS to test for the LF antigen.

### 2.5. Investigation of the Intensity of Microfilariae Infection

Any participants who tested positive on the FTS had further blood samples (100 µL) taken that evening of the day of sampling between 22:00 hours and 02:00 hours to detect the presence of *W. bancrofti* microfilariae. The blood samples were taken at night because of the microfilariae behavior of nocturnal periodicity, whereby in the day, the microfilariae are in the deeper blood vessels, and at night, they are in the superficial capillaries. Therefore, for this purpose, samples were commonly taken between 22:00 hours and 02:00 hours. 

This was arranged with the individual at the time of results feedback, informing them of their FTS result. In this traditional gold standard technique, blood smears for the detection of microfilariae required the collection of 100 µL of blood from each participant were clearly labeled and stored in 1.5 mL vials until further analysis in the laboratory. In the laboratory, the blood samples were evaluated for microfilariae intensity using the Sedgewick Rafter counting chamber. After examining the blood using the counting chambers, some of it was applied onto a slide, dried, and stained (with Giemsa) before being examined [19,20].

### 2.6. Questionnaire

A questionnaire capturing information on the individuals tested and whether they had participated in the MDA or not was carried out and results were recorded using an Open Data Kit (ODK) on an Android tablet and phone. The data records were geographical positioning system (GPS) enabled and field FTS results were photographed.

### 2.7. Statistical Analysis

Univariate and bivariate analyses were done using a Statistical Package for the Social Sciences (SPSS). Pearson’s uncorrected Chi-square test was used to compare proportions at a 5% significance level. Associations were established disregarding the ‘Don’t know’ category.

### 2.8. Managing the Positive Cases

The *Wb* Ag-positive cases that were detected during the study were treated with a single dose of a combination of Albendazole (400 mg) plus diethylcarbamazine (6 mg/kg). This treatment regime is because no mapping nor investigations have been done to determine the endemicity of Onchocerciasis caused by *Onchocerca volvulus* (River blindness), and thus Zambia is said to be non-endemic for Onchocerciasis. Therefore, the drugs used for treating individual *Wb* Ag-positive cases and for MDA against LF are Diethylcarbamazine (DEC) and Albendazole (ALB) [21]. The associated DEC adverse drug reaction (ADR) that could arise from Onchocerciasis treatment as the country treats LF cases remains undocumented since the mapping of Onchocerciasis is yet to be conducted in Zambia. Additionally, there are problems in using LF drugs when Onchocerciasis is present because DEC is not effective in killing *Onchocerca volvulus,* the parasite that causes Onchocerciasis [22].

## 3. Results

A total of 47,235 participants were tested and interviewed from 148 sites (70 sentinel and 78 spot-check sites). Of these, valid tests were 47,052, of which 27,762 (59%) were females and 19,290 (41%) were males. Most (62.3%) of the people sampled were older than 15 years. The positivity rate for *W. bancrofti* antigenaemia (*Wb* Ag) was 0.14%.

**Figure 1:**
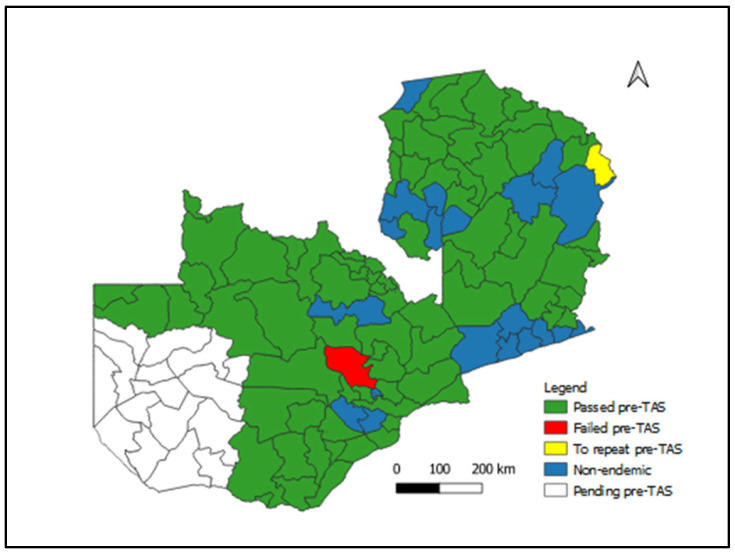
Lymphatic Filariasis Status of pre-TAS Implementation by District, Zambia, 2022.

Figure 1 shows LF *Wb* Ag prevalence by district conducted in 2021 and 2022. The pre-TAS showed that 78 districts passed the pre-TAS and are currently due for transmission assessment survey (TAS) 1. Passed pre-TAS means less than 2% antigenemia; Failed pre-TAS means antigenemia > 2% Ag detected.

The number of evaluation units was 80 districts with 47,235 (98.4%) out of the targeted 48,000 tests conducted. There were 31 (38.8%) evaluation units that had *Wb* Ag-positive individuals (Table 1). Mafinga district will conduct pre-TAS again, as the exercise was not complete.

The overall prevalence of *Wb* Ag was 0.14% (Table 2). The *Wb* Ag prevalence was higher (0.2%) in male than female participants. Regarding age, the older age group above 15 years of age had a higher (0.16%) prevalence of *Wb* Ag than the age group of less than 15 years which had a prevalence of 0.11%. The highest provincial *Wb* Ag prevalence was in Central Province at 0.29% while the lowest provincial *Wb* Ag prevalence was in Lusaka Province at 0.03%

About 61.5% and 73.8% of the participants aged less than 15 years and above 15 years, respectively, received MDA (Table 3). Significantly (*p* < 0.001), more older participants received MDA than younger participants. More female participants at 71.3% than male participants at 65.9% received MDA which was a statistically significant finding (*p* < 0.001). The *Wb* Ag positivity amongst the participants that received MDA was not related to the number of rounds or doses (*p* = 0.579).

The positivity amongst participants that received MDA was 0.14% and amongst participants that did not receive MDA, it was also 0.14% (Table 4). Therefore, there was no statistically significant difference (*p* = 0.803) in the proportion of Wb Ag positivity between the two groups.

## 4. Discussion

It has been shown and therefore accepted that LF can be eliminated as a public health problem after a minimum of five effective rounds of MDA and demonstrating low prevalence [23]. The first assessments recommended by WHO are known as pre-TAS, and are conducted in each implementation unit after five effective rounds of MDA [24,25]. All IUs should have had at least five effective (≥65%) rounds of MDA and in all sentinel and sport-check sites, the prevalence of Mf should be <1% or the Wb Ag should be <2% at all sites after the last effective round. Failure to pass pre-TAS means that further rounds of MDA are required. 

Following the successful conducting of five rounds of LF MDA, Zambia went ahead and conducted a pre-TAS. The survey revealed in the 79/80 endemic districts a prevalence of *Wb* Ag of 0.14%; overall, this *Wb* Ag prevalence is less than the target of 2% and 0.0% prevalence of Mf, which is within the target of less than 1%. Of the districts surveyed, only Chibombo had a *Wb* Ag prevalence greater than 2%. This is the only district whose *Wb* Ag prevalence failed to meet the threshold of less than 2% antigenemia for an EU to pass a pre-TAS. This could then suggest that all the surveyed districts, except for Chibombo, passed pre-TAS. This further implies that there is a need to conduct TASs in these districts in order to decide whether to stop MDA or not. The results also indicate that in Zambia, Chibombo is the only district where MDA should be continued. When a country or an IU fails to meet the established thresholds in a pre-TAS, at least two more rounds of MDA must be implemented.

The finding in this study that 65 cases which were positive for circulating filarial antigens (CFA) were negative for microfilaria (as microfilaria could not be found in their blood) was not surprising. It is possible for individuals infected by filarial worms to be either microfilaraemic or amicrofilaraemic, depending on whether microfilariae can be found in their peripheral blood [26,27,28]. Filariasis is diagnosed in microfilaraemic cases primarily through direct observation of microfilariae in the peripheral blood. 

The results showed that more women were interviewed than men. Similarly, more females participated in MDA than men. The reason for this could partly be due to the fact that the surveys were done at the beginning of the rainy season (December to January) and so most men could have been busy preparing their crop (maize) fields. The other reason is that, generally, when you examine census population figures in Zambia, there are more women than there are men [29]. Thus, any given sample is bound to have more females than men as a result of this skewed population structure.

It is interesting to note that of the participants tested, more were older than 15 years. This may mean that the older age group paid more attention to the pre-TAS exercise. The majority of tested participants were those with one and two rounds of MDA. This may mean that not all people have had all five rounds of MDA. Even if the MDA coverages in most of these districts were above 65% as per the WHO threshold for achieving elimination, not everyone participated in every MDA campaign. However, it was interesting to note that those that had one or five rounds of MDA were not found to have circulating filarial antigens of *Wb* Ag [30].

These results are encouraging and suggest that the LF MDA programme has contributed to reducing the prevalence of circulating microfilaria in the endemic districts. However, it may be difficult to attribute the reduced prevalence solely to the success of the LF MDA programme [31]. This could be so because the other interventions that target mosquitos such as the distribution of insecticide-treated mosquito nets (ITNs), as was the case with the Rollback Malaria Programme, could have greatly impacted our findings. This is for the simple reason that ITNs play a significant role (if used correctly and consistently) in reducing mosquito bites, thereby reducing the transmission of the parasite as it is equally transmitted by mosquitoes [32].

That said, it can be concluded that the LF MDA in Zambia has had a great impact as it has managed to reduce the prevalence of LF to almost zero and that LF MDA may no longer be necessary in 78 of the 80 LF endemic districts of Zambia. Chibombo is the only district that requires LF MDA. Chibombo district lies along a busy and main transport route between Lusaka Capital City of Lusaka Province and Kabwe City of Central Province. The district has no international borders. While Chibombo is more of a farming district, there is high population mobility. According to Buckley, it is possible for individuals from other areas to be tested and found positive while living or visiting other areas.

It also suffices to state that this success has probably resulted from a concerted effort by all the stakeholders involved. It is, therefore, recommended with the results of this pre-TAS from these nine provinces to suggest that Zambia should move into conducting the TASs to ascertain if MDA should be stopped or not.

The LF pre-TAS follows a prototype design and hence no details of other interventions were included and there is guidance on the sample size of 600 participants in each district or evaluation unit.

## 5. Conclusions

There is a significant decline in LF transmission in Zambia after five effective rounds of MDA. The country qualifies to conduct TAS 1 in 78 districts of the 80 endemic districts.

## Figures and Tables

**Table 1:** Number of endemic districts and tests conducted by province (*n* = 47,235).

Province	Number of Endemic Districts	Districts with +ve *Wb* Ag	Tests	Valid Tests	% Valid Tests	Invalid Tests	% Invalid Test
Central	12	7	7358	7317	99.44	41	0.56
Copperbelt	9	2	5044	5042	99.96	2	0.04
Eastern	7	4	4176	4176	100.00	0	0.00
Luapula	6	5	3587	3550	98.97	37	1.03
Lusaka	6	1	3674	3670	99.89	4	0.11
Muchinga	6	2	2869	2828	98.57	41	1.43
N/Western	11	3	6523	6523	100.00	0	0.00
Northern	12	5	7352	7303	99.33	49	0.67
Southern	11	2	6652	6643	99.86	9	0.14
Zambia	80	31	47,235	47,052	99.61	183	0.39

**Table 2:** Prevalence of *Wb* Ag by sex, age, and province (*n* = 47,052).

Characteristics		Total	Positive (%)	(95% CI)	*p*-Value
Total		47,052	65 (0.14)	0.14 [0.11, 0.18]	
Sex					
	Female	27,762	26 (0.09)	0.09 [0.06, 0.14]	0.002
	Male	19,290	39 (0.20)	0.20 [0.14, 0.28]	
Age	(Years)				
	≤15	17,750	19 (0.11)	0.11 [0.06, 0.17]	0.158
	>15	29,302	46 (0.16)	0.16 [0.11, 0.21]	
Province					
	Central	7317	21 (0.29)	0.29 [0.18, 0.44]	<0.001
	Copperbelt	5042	3 (0.06)	0.06 [0.02, 0.17]	
	Eastern	4176	6 (0.14)	0.14 [0.05, 0.31]	
	Luapula	3550	10 (0.28)	0.28 [0.15, 0.52]	
	Lusaka	3670	1 (0.03)	0.03 [0.00, 0.01]	
	Muchinga	2828	3 (0.11)	0.11 [0.02, 0.31]	
	N/Western	6523	4 (0.06)	0.06 [0.02, 0.16]	
	Northern	7303	15 (0.22)	0.22 [0.11, 0.34]	
	Southern	6643	2 (0.03)	0.03 [0.00, 0.11]	

**Table 3:** Participation in MDA according to age, sex, and MDA rounds.

Age (Years)	Total	Don’t Know (%)	No (%)	Yes (%)	*p*-Value
≤15	17,750	305 (1.7)	6534 (36.8)	10,911 (61.5)	<0.001
>15	29,302	128 (0.4)	7563 (25.8)	21,611 (73.8)	
Total	47,052	433 (0.9)	14,097 (30.0)	32,522 (69.1)	
Sex	Total	Don’t know (%)	No (%)	Yes (%)	*p*-value
Female	27,762	264 (1.0)	7697 (27.7)	19,801 (71.3)	<0.001
Male	19,290	169 (0.9)	6400 (33.2)	12,721 (65.9)	
Total	47,052	433 (0.9)	14,097 (30.0)	32,522 (69.1)	
MDA Round	Total received MDA.	Proportion received MDA (%)	Number of *Wb* Ag + ve cases	Prevalence (%)	*p*-value
1	13,482	41.46	0	0.00	0.579
2	12,304	37.83	17	0.13	
3	5036	15.48	17	0.34	
4	1027	3.16	10	0.97	
5	673	2.07	0	0.00	
	32,522	100.00	44	0.14	

**Table 4:** *Wb* Ag positivity by MDA (*n* = 47,052).

	Valid Tests	*Wb* Ag. + ve	*Wb* Ag. + ve%	*p*-Value
Ever received MDA	32,522	44	0.14	0.803
Did not receive MDA	14,530	21	0.14	
	47,052	65	0.14	

## Data Availability

Data is available on request.
